# mGPDH Deficiency leads to melanoma metastasis via induced NRF2

**DOI:** 10.1111/jcmm.16542

**Published:** 2021-05-03

**Authors:** Xing Li, Ling Zhou, Yiming Zhang, Xuan He, Hao Lu, Linlin Zhang, Yongfeng Tian, Xiufei Liu, Hongting Zheng, Jiaqing Shao, Min Long

**Affiliations:** ^1^ Department of Endocrinology Translational Research Key Laboratory for Diabetes Xinqiao Hospital Army Medical University Chongqing China; ^2^ Department of Endocrinology Jinling Hospital, Medical School of Nanjing University Nanjing China; ^3^ Department of Plastic and Cosmetic Surgery Xinqiao Hospital Army Medical University Chongqing China; ^4^ Department of Cancer Center Daping Hospital Army Medical University Chongqing China; ^5^ Department of Dermatology Chongqing Traditional Chinese Medicine Hospital Chongqing China

**Keywords:** melanoma, metastasis, mGPDH, NRF2

## Abstract

Oxidative stress critically influences carcinogenesis and the progression of melanoma, and aggressive malignant melanoma activity is due to its high metastatic ability. Some findings in several cancer cell lines have indicated that mGPDH, a component of the mitochondrial respiratory chain, also modulates oxidative stress. However, the role of mGPDH in melanoma remains elusive. Here, we report that the mGPDH protein level is decreased in human skin melanoma compared to normal skin and decreased in metastatic melanoma compared to primary melanoma. Our in vivo and in vitro experiments indicated that mGPDH depletion accelerated melanoma migration and invasion without affecting proliferation or apoptosis. Mechanistically, we found elevated NRF2 protein levels in human skin melanoma and mGPDH‐knockout (ko) metastatic xenografts in the lungs of nude mice. Moreover, in A375 melanoma cells, the loss of mGPDH‐induced NRF2 expression but did not affect NRF2 protein degradation. Additionally, melanoma metastasis induced by the loss of mGPDH was rescued by the further down‐regulation of NRF2 in vivo and in vitro. Consistently, mGPDH overexpression (oe) depressed NRF2 expression and attenuated the malignant properties of melanoma cells. In conclusion, our findings suggest that mGPDH suppresses melanoma metastasis by inhibiting NRF2 and downstream oxidative signals, highlighting the therapeutic potential of mGPDH for melanoma treatment.

## 1 INTRODUCTION

Melanoma, which is also known as malignant melanoma, is among the most aggressive forms of skin cancer and has a poor cure rate due to its invasive nature.^1^, ^2^ Melanoma mostly develops from pigment‐containing cells known as melanocytes and thus predominantly a disease of the skin but can also occur at mucous membranes and other sites.^3^ With treatment, in the United States, the 5‐year survival rate is 65% among patients with cancer that has spread to the lymph nodes and 25% among those with cancer with distant metastasis.^2^ In recent decades, cumulative evidence, including that our data,^4^ has indicated that oxidative stress critically influences carcinogenesis and the progression of cancer, especially tumour metastasis. Furthermore, redox imbalance plays a central role in the genesis and development of melanoma.^5^

A promising molecule for regulating the cellular antioxidant response is mitochondrial glycerol‐3‐phosphate dehydrogenase (mGPDH),^6^ which was previously considered mainly an integral component of the respiratory chain. In addition to stimulating glycerol production to promote obesity,^7^ mGPDH regulates the oxidative phosphorylation (OXPHOS) rate and is effectively targeted by metformin.^8^, ^9^ Notably, recent research has shown that mGPDH can modulate cell growth in thyroid cancer^8^ and reactive oxygen species (ROS) generation in the oxidative stress‐induced progression of prostate cancer and that mGPDH functions as a crucial regulator of mitochondrial oxidative stress.^10^, ^11^, ^12^ Oxidative stress and consequent oxidative damage are important contributors to tumour metastasis.^13^ Our previous studies further demonstrated that nuclear factor (erythroid‐derived‐2)‐like 2 (NRF2, also known as NFE2L2) modulates cell migration in both normal skin cells^14^ and cancer cells.^4^ Accumulating evidence has established that the NRF2 pathway plays a major role in the cellular antioxidant response.^15^ Currently, NRF2 is considered a hallmark of cancer with both tumour‐suppressive and tumour‐promoting effects.^4^, ^16^ Thus, does mGPDH contribute to tumour metastasis, especially in malignant tumours with highly invasive characteristics such as melanoma? During pathogenesis and development, does mGPDH modulate oxidative stress, potentially via NRF2? So far, studies investigating the role of mGPDH in melanoma, especially in the progression of metastasis of this cancer, are lacking.

In this study, we determined mGPDH expression in normal skin and melanoma tissues from patients. A tissue array containing numerous human tissues from primary and metastatic melanoma at various stages revealed a correlation between mGPDH and melanoma progression. In vivo and in vitro experiments using stable short hairpin RNA (shRNA)‐transfected cells, nude mouse xenograft experiments and living cell fluorescence signal analysis demonstrated that mGPDH deficiency leads to melanoma cell migration and invasion as a result of activation of the endogenous antioxidant stressNRF2. We further observed that the inhibition of NRF2 had an antitumour effect on low mGPDH‐induced melanoma metastasis. These results have uncovered the role of mGPDH in melanoma metastasis and suggest the potential use of targeted therapies to treat melanoma with low mGPDH expression levels.

## 2 MATERIALS AND METHODS

### 2.1 Patients and samples

The collection of skin melanoma and adjacent normal skin tissues from three patients was approved by the Medical Ethics Committee of the Second Affiliated Hospital of the Army Medical University (No. 2018‐096‐01). Melanoma epidermal skin layers and normal skin were dissected into 7‐mm pieces and stored at −80°C. The clinical characteristics of the patients are shown in Table 1.

**TABLE 1:** Clinical patients’ information

	Sex	Age	Tumor region	Clinical diagnosis	Characteristics	Positive IHC marker
Patient 1	Female	47	Plantar skin	Maligment melanoma	1.5 × 1 cm, black crust, clear boundary	S100, HMB45, Ki67, MelanA, Vimentin
Patient 2	Female	50	Sacrococcygeal skin	Maligment melanoma	4 × 4 cm, Ulcerated black crust	S100, Desmin, SMA, Ki67, CD117
Patient 3	Male	63	Plantar skin	Maligment melanoma	1.5 × 1.5 cm, Ulcerated nevus spilus,	S100, HMB45, Ki67, MelanA

### 2.2 Immunohistochemistry and H&E staining

Human melanoma tissue microarray slides corresponding to tissues from 101 patients with primary malignant melanoma and 53 patients with metastatic malignant melanoma (lymphatic metastasis) (ME2082c) were obtained from US Biomax (Derwood). The slides were fixed in 4% PFA (Invitrogen) and dehydrated with an ethanol concentration gradient. Then, the slides were stained with anti‐mGPDH antibody (Abcam). Immunohistochemical (IHC) staining was performed according to the standard protocol.^17^ The tumour cell morphology was examined under a microscope (Leica Microsystems). The IHC staining results were scored independently by two pathologists to determine the percentage of positive cells (five scores: 0 [10%], 1 [10%‐25%], 2 [25%‐50%], 3 [50%‐75%] and 4 [75%‐100%]) and staining intensity (four scores [from low to high]: 0, 1, 2 and 3), shown as representative IHC images in Figure S1A. The final IHC staining score was determined by the following equation: IHC score = intensity score × percentage score. Mouse tissue sections were fixed in 10% buffered formalin and embedded in paraffin. The tissue sections (5 μm) were stained with haematoxylin and eosin (H&E) and then subjected to standard deparaffinization. Sample information and the mGPDH IHC scores are listed in Table S1.

### 2.3 Cell culture, antibodies and other reagents

A375 human malignant melanoma cells were obtained from the Zhong Qiao Xin Zhou Biotechnology Corporation. The cell line was authenticated by short tandem repeat analysis with GeneMapper software. The cells were grown in Dulbecco's modified Eagle's medium (DMEM) supplemented with 10% foetal bovine serum (FBS) at 37°C in a 5% CO_2_ air atmosphere. DMEM, FBS, 100× penicillin and streptomycin were acquired from Gibco. Antibodies against the following proteins were used: mGPDH (sc‐390830), NRF2 (sc‐13032, sc‐722), haem oxygenase 1 (HO‐1) (sc‐10789), cyclooxygenase (COX‐2) (sc‐166475) and β‐actin (sc‐47778) (Santa Cruz).

### 2.4 siRNA and plasmid transfection

Transfection with small interfering RNA (siRNA) and plasmids was performed with RNAiMAX or Lipofectamine 3000 (Invitrogen) according to the manufacturer's instructions. mGPDH siRNA, control siRNA, mGPDH plasmid and the vector were obtained from Ruibo Genetech. The siRNA and plasmid sequences are listed in Table S2.

### 2.5 Construction of a stable shRNA‐expressing melanoma cell line

Human mGPDH shRNA, control shRNA (ubi‐MCS‐firefly‐Luciferase‐IRES‐Puromycin), sh‐ko‐control, sh‐ko‐mGPDH, sh‐oe‐control and sh‐oe‐mGPDH were purchased from the GeneChem Company. The sequences are listed in Table S2. Treated 96‐well cell culture plates and DMEM containing 10% foetal calf serum were used. The cells reached 40% confluence before shRNA transduction. Two microlitres of lentiviral particles were added per well, and the cells were incubated for 6 hours before the medium was changed. After one day of maintenance, the cells that were illuminated after the addition of luciferin (Beyotime) under a bioluminescence imaging system were chosen.

### 2.6 Cell migration and invasion

Cellular Transwell assays were used to test cell migration and invasion. Cells (2 × 10^4^/well) were inoculated into serum‐free medium in the inserts of Transwell^®^ cell culture chambers (8 mm pore size; Corning) to assess cell migration and invasion. The lower chamber was filled with DMEM containing 20% FBS. The mean numbers of cells in five randomly chosen fields per well were compared between groups. For Matrigel invasion assays, inserts with an 8 µm pore size were coated with 1 mg/mL Matrigel (BD Bioscience) for 24 hours. Cells were incubated for 24 hours and allowed to migrate or invade. Cells that migrated to or invaded the lower surface of the membrane were fixed with 4% PFA and then stained with crystal violet as previously described. Five random fields per chamber were counted using an inverted microscope.

### 2.7 *In* *vivo* tumour growth and metastasis

To examine in vivo tumour growth, control or mGPDH‐knockout (ko) shRNA‐injected stable A375 cells were subcutaneously injected into nude mice. Every 2 days for 2 weeks, tumour sizes were measured with a calliper, and the tumour volumes were calculated using the following formula: V = (4/3) × π × (L/2) × (L/2) × (D/2), where V is the tumour volume, W is the tumour width, L is the tumour length and D is the tumour depth.^18^ At the end of the experiment, the mice were sacrificed, and the tumours were excised and weighed. For in vivo metastasis experiments, mGPDH‐ko or control shRNA‐Luciferase, Sh‐control A375, Sh‐ko‐mGPDH, Sh‐ko‐mGPDH Sh‐ko‐NRF2, Sh‐overexpression (oe)‐mGPDH or control A375 cells were intravenously injected into nude mice. At 8 weeks post‐injection, bioluminescence imaging was conducted to measure the tumour burden in vivo. The mice were anaesthetised with 1.5% isoflurane and intraperitoneally injected with a 150 mg/kg D‐luciferin solution (diluted in DPBS without Mg^2+^ or Ca^2+^) in the left lower quadrant. Bioluminescence images were acquired with an in vivo imaging system (IVIS; Caliper Life Sciences). The mice were sacrificed after imaging, and the lungs were isolated, fixed or preserved by freezing. All animal protocols had been approved by the Army Medical University Institutional Animal Care and Use Committee.

### 2.8 Quantitative real‐time PCR (qRT‐PCR)

Total RNA was extracted from the harvested cells, and 1 µg of RNA was reverse transcribed into cDNA as described above. qRT‐PCR was performed with SYBR Premix Ex Taq II (Takara, Terra Bella Ave) using an Applied Biosystem 7300 system (Thermo Fisher Scientific). The primer sequences are listed in Table S3.

### 2.9 Western blot (WB) analysis

Immediately following dissection, the melanoma and skin tissue samples were rinsed with cold PBS and placed in a tube on ice. Small tissue samples (200 mg) were chopped with clean dissecting instruments. From cells, cell lysates were prepared in ice‐cold lysis buffer. Proteins (40 μg/lane) were separated by gel electrophoresis on 10% SDS‐PAGE gels. The separated proteins were transferred onto PVDF membranes (Bio‐Rad, Hercules) by electroblotting at 100 V for 90 minutes. The membranes were probed with the respective primary antibody, followed by incubation with an HRP‐conjugated secondary antibody and detection by chemiluminescence. The primary antibodies were used at 1:800‐1:1000 dilution, and the secondary antibodies were used at 1:3000 dilution.

### 2.10 Determination of the NRF2 half‐life

Cells were treated with mGPDH siRNA or control siRNA for 48 hours. To block protein synthesis, cycloheximide (CHX, 50 μmol/L, BioVision) was added, and cell lysates were collected every 15‐min after CHX treatment and subjected to immunoblotting with anti‐NRF2 antibody.^19^ The relative band intensities were quantified using the Fusion FX5s system (Vilber Lourmat), and ImageJ software was used to analyse the half‐life values.

### 2.11 Apoptosis assay

Cell apoptosis was evaluated with an Annexin V/PI kit (BD Biosciences). Cells were seeded into 6‐well plates at a concentration of 5 × 10^5^/mL after siRNA treatment for 48 hours. The cells were washed with PBS and incubated with Annexin V/PI at 20‐25°C for 25 minutes, and cell apoptosis was measured with a fluorescence‐activated cell‐sorting flow cytometer (Beckman Coulter Gallios).

### 2.12 Detection of the ATP content

Cellular ATP levels were determined using an ATP assay kit (Beyotime) following the manufacturer's instructions. Cells were lysed with 200 μL of cell lysis reagent (Beyotime) and then quantified. Luciferase reagent (1 μL) and dilution buffer (100 μL) were added to each well. After 3 minutes, 50‐μL lysate samples were added. Then, the luminescence was measured with a luminometer.

### 2.13 Statistical analyses

The data are presented as the mean ± standard deviation (SD), frequency or percentage. Statistical significance was determined using Student's *t* test, one‐tailed Fisher's exact test, or one‐way ANOVA with Newman‐Keuls multiple comparisons test. The statistical analysis software GraphPad Prism (6.01) was used for statistical analyses, and *P* < 0.05 indicated statistical significance.

## 3 RESULTS

### 3.1 mGPDH expression was down‐regulated in melanoma

**FIGURE 1:**
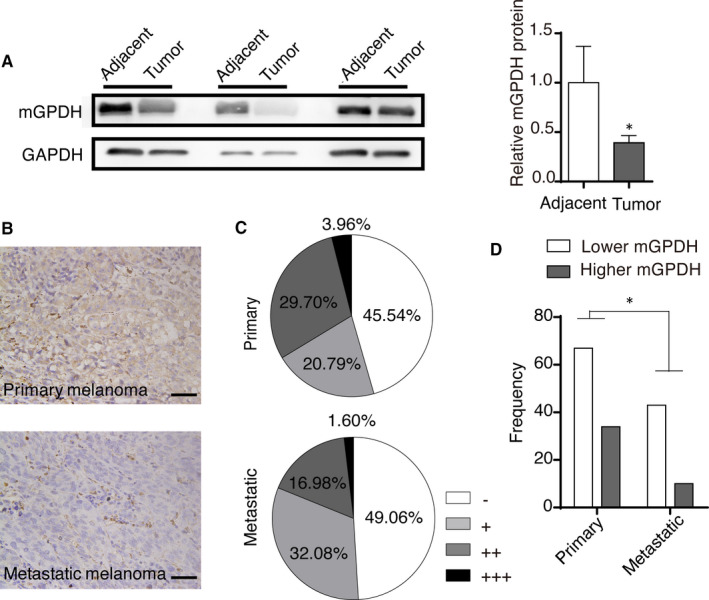
Decreased mGPDH expression in primary and metastatic melanoma samples. A, mGPDH protein levels and WB grey value quantification in skin melanoma tissue and adjacent skin tissue from 3 patients (n = 3). B‐D, mGPDH protein expression in a commercial melanoma tissue array (ME2082c, Biomax) was detected by IHC. The tissue array contained tissue from 101 patients with primary malignant melanoma (diagnosed with stage I in 6 cases, II in 55 cases, III in 8 cases and IV in 2 cases; T1 in 3 cases, T2 in 11 cases, T3 in 4 cases and T4 in 53 cases; and N0 in 63 cases, N1 in 7 cases and N2 in 1 case; containing 45 skin, 16 recta, 6 eye and 6 soft tissue samples) and 53 metastatic malignant melanoma tissues (lymphatic metastasis). B, Representative IHC images of mGPDH in primary and metastatic melanoma tissue sections. Scale bar, 50 μm. C, The distribution of mGPDH IHC scores for the tissue sections from the primary and metastatic melanoma groups. IHC scores: ‐: 0‐2; +: 2‐4; ++: 4‐8; +++: 8‐12. D, The frequencies of tissue sections with high mGPDH expression (IHC score≥4) and low mGPDH expression (IHC score<4) in the primary and metastatic melanoma groups. In A, the *p*‐value was derived from Student's *t* test; In D, the *P*‐Values were derived from one‐tailed Fisher's exact tests. *P < 0.05

To investigate the possible association between mGPDH and melanoma, we first observed mGPDH protein expression in melanoma tissues. Our results showed that the protein level of mGPDH was significantly decreased in human melanoma skin tissue compared to adjacent normal skin (Figure 1A). With the melanoma tissue array, we found that most primary melanoma tissues showed more obvious IHC staining for mGPDH than the metastatic tissues, as shown in Figure 1B. Specifically, the percentage of primary melanoma patients with a high IHC score (++, +++) was 33.66%, which was more than that for patients with metastatic melanoma (18.58%) (Figure 1C). Consistently, after sorting the melanoma tissues into high and low mGPDH expression groups, the Fisher's exact test further confirmed the significant correlation between mGPDH expression and the status of the melanoma as primary or metastatic (Figure 1D). In addition, even in the primary melanoma tissue, the percentage of samples from more severe tumours (stages III and IV) negative for mGPDH staining was higher than that among samples from less severe tumours (stages I and II) (Figure S1B), and similar mGPDH expression profiles were found in primary melanoma tissues with lymph node metastasis (Figure S1C). In summary, down‐regulated mGPDH expression was observed in melanoma tissues from patients and metastatic and primary melanoma tissues at higher tumour stages in the human tissue array. These results indicate the correlation of mGPDH and melanoma and suggest the potential involvement of decreased mGPDH expression in melanoma progression.

### 3.2 mGPDH silencing induced melanoma cell metastasis in vitro and in vivo

To assess the possible role of mGPDH in the progression of melanoma, loss‐of‐function studies were performed in vitro and in vivo. First, a proliferation assay did not show a significant difference in proliferation between A375 cells transduced with control siRNA and those transduced with mGPDH siRNA (Figure [Link], [Link]). To comprehensively assess the effect of mGPDH on tumour growth in vivo, nude mice were subcutaneously injected with stable cell lines transduced with control shRNA or mGPDH‐ko shRNA. Over 14 days of continuous observation, neither the tumour weight nor tumour volume was significantly different between the two groups of mice (Figure [Link], [Link]), suggesting that mGPDH silencing did not influence melanoma proliferation in vivo or in vitro. In a subsequent exploration of the role of apoptosis in the effect of mGPDH, the percentage of Annexin V‐positive cells did not show a significant change following mGPDH expression loss (Figure S3A,B). Moreover, mGPDH silencing did not affect ATP production (Figure [Link], [Link]).

**FIGURE 2:**
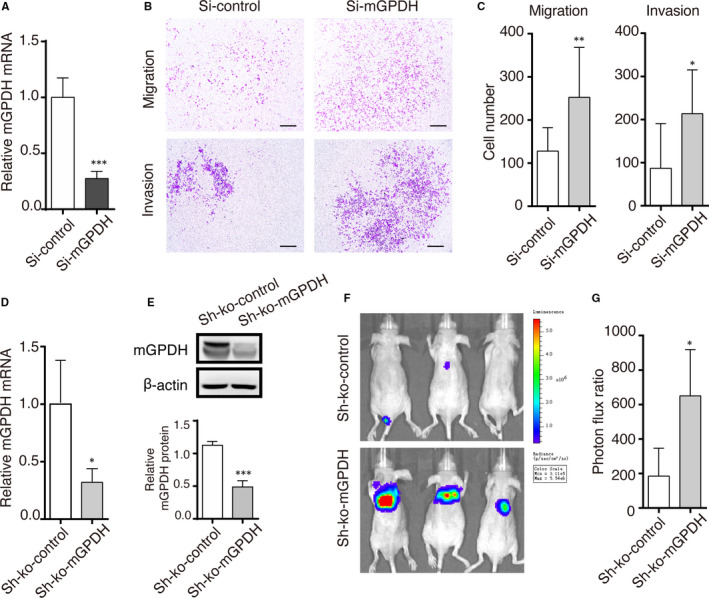
mGPDH silencing induced melanoma cell metastasis in vitro and in vivo. A, RT‐PCR validation of the knockdown efficacy of siRNA targeting mGPDH. B, Transwell migration and Matrigel invasion assays of A375 cells transfected with control siRNA or mGPDH siRNA. Scale bar, 200 μm. C, Results of cell quantification by Transwell migration and Matrigel invasion assays (n = 3). D‐G, Luciferase sh‐control shRNA (Sh‐ko‐control) and mGPDH‐ko shRNA (Sh‐ko‐mGPDH) were transfected into A375 cells to construct stable melanoma cell lines. The cells (2 million) were injected into nude mice via the tail vein, and melanoma cell metastasis was observed in vivo. Transfected A375 cell lines were continuously cultured for 8 wk to ensure consistency with the timing of the in vivo experiment. D. Relative mRNA expression (n = 3). E, Protein levels of mGPDH were detected by intensity quantification. F, G, In vivo metastasis assays. At 8 wk post‐injection of the stable cells, melanoma cell metastasis in nude mice was assessed by bioluminescence imaging, and the photon flux ratio was quantified (n = 5‐7 mice per group). In A, C, D and G, the *P‐*values were derived from Student's *t* tests. *P < 0.05, **P < 0.01, ***P < 0.001

To detect the effect of mGPDH on melanoma cell metastasis, in vitro migration and invasion assays were conducted. A375 cells transduced with mGPDH, siRNA expressed significantly decreased levels of mGPDH (Figure 2A) and displayed increased mobility, including exaggerated migration and invasion, as detected by the Transwell assay (Figure 2B,C). Moreover, in vivo tumour xenograft assays with mGPDH‐ko cells confirmed by qPCR and WB analysis (Figure 2D,E) were performed in immunodeficient nude mice using A375 cells transduced with shRNA tagged with luciferase to facilitate the study of xenograft‐derived tumours. The in vivo luminescence of the tumours showed that melanoma metastasis was mainly observed in the lungs and that the luminescence from the mGPDH‐ko‐A375 xenografts was less intense with a higher photon flux ratio (Figure 2F,G). Taken together, the above results demonstrate that mGPDH depletion is essential for the distant colonization of melanoma cells and promotes the metastasis of melanoma.

### 3.3 mGPDH silencing activated the NRF2 pathway in melanoma

**FIGURE 3:**
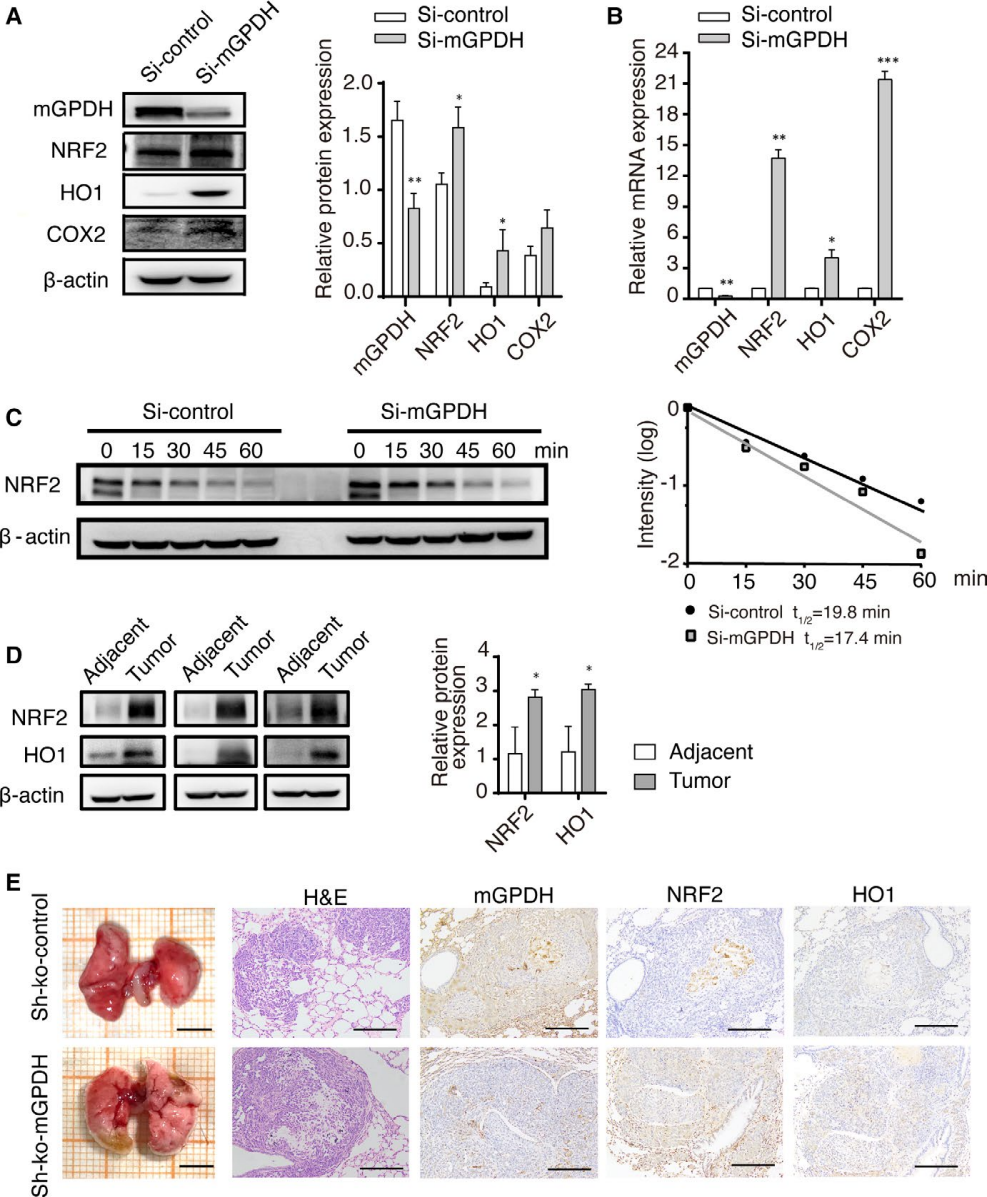
mGPDH silencing activated NRF2. A, B, The protein and the mRNA levels of mGPDH and NRF2 signalling pathway in A375 cells without or with mGPDH siRNA treatment were detected by WB analysis and RT‐PCR (n = 3). C, NRF2 protein half‐life (t_1/2_) in A375 cells transduced with mGPDH siRNA was determined by pulse‐chase assay and immunoblotting. A375 cells were transfected with control siRNA and mGPDH siRNA for 48 h, after which cycloheximide (50 μmol/L) was administered to block protein synthesis. D. Protein levels of NRF2 and HO‐1 in skin melanoma tissue and adjacent skin tissue from patients were determined by intensity quantification (n = 3). E. Lung tissues of melanoma metastasis from nude mice injected with Luciferase‐control shRNA (Sh‐ko‐control)/mGPDH‐ko shRNA (Sh‐ko‐mGPDH) A375 cells were harvested. Representative images of lungs and their corresponding tissue sections stained with H&E are shown. Scale bars, 500 μm (gross morphology) and 200 μm (H&E staining). The expression of mGPDH, NRF2 and HO‐1 was detected by IHC, as shown in the right three images. Scale bar, 200 μm. The *P*‐Values were derived from Student's *t* tests. *P < 0.05, **P < 0.01, ***P < 0.001

mGPDH contributes to the homeostasis of mitochondrial oxidative stress,^8^ which is also critically maintained by the endogenous antioxidative NRF2 pathway.^20^ Interestingly, previous studies established that NRF2 activation in melanoma cells induces tumour metastasis^4^ and that NRF2 acts as a driver of cancer progression.^20^ Thus, we detected members of the NRF2 pathway after mGPDH silencing. Our data showed significantly decreased mGPDH protein and mRNA levels and increased expression of NRF2 and the downstream signalling molecules HO‐1 and COX‐2 in A375 cells transduced with mGPDH siRNA (Figure 3A,B). Since the elevated NRF2 protein expression could have been a result of increased NFE2L2 gene transcription and/or decreased NRF2 protein degradation,^21^, ^22^ we measured the NRF2 protein half‐life (t_1/2_) by 15‐min interval sampling after CHX treatment, which showed no significant difference in NRF2 protein half‐life between A375 cells transduce with mGPDH siRNA (t_1/2_ = 17.4 minutes) and control siRNA (t_1/2_ = 19.8 minutes) (Figure 3C). Consistent with the results of the in vitro experiment, we observed that NRF2 and HO‐1 protein levels were significantly higher in skin melanoma tissues from the patients compared to adjacent normal tissues (Figure 3D). Similarly, in a nude mouse model of melanoma metastasis, lungs from mice with mGPDH‐ko xenografts were consolidated and exhibited more metastases, and their surface appeared haemorrhagic and granulated, unlike the lung surface of mice with control A375 xenografts (Figure 3E). Moreover, the lung tissue samples were histologically processed and stained with anti‐human anti‐mGPDH, anti‐NRF2 and anti‐HO‐1 antibodies (Figure 3E). Consistent with the above results, the mGPDH‐ko A375 xenografts expressed elevated protein levels of NRF2 and HO‐1. Therefore, our data suggest that mGPDH silencing activates the NRF2 pathway in melanoma.

### 3.4 Down‐regulation of NRF2 rescued mGPDH loss‐induced melanoma metastasis

**FIGURE 4:**
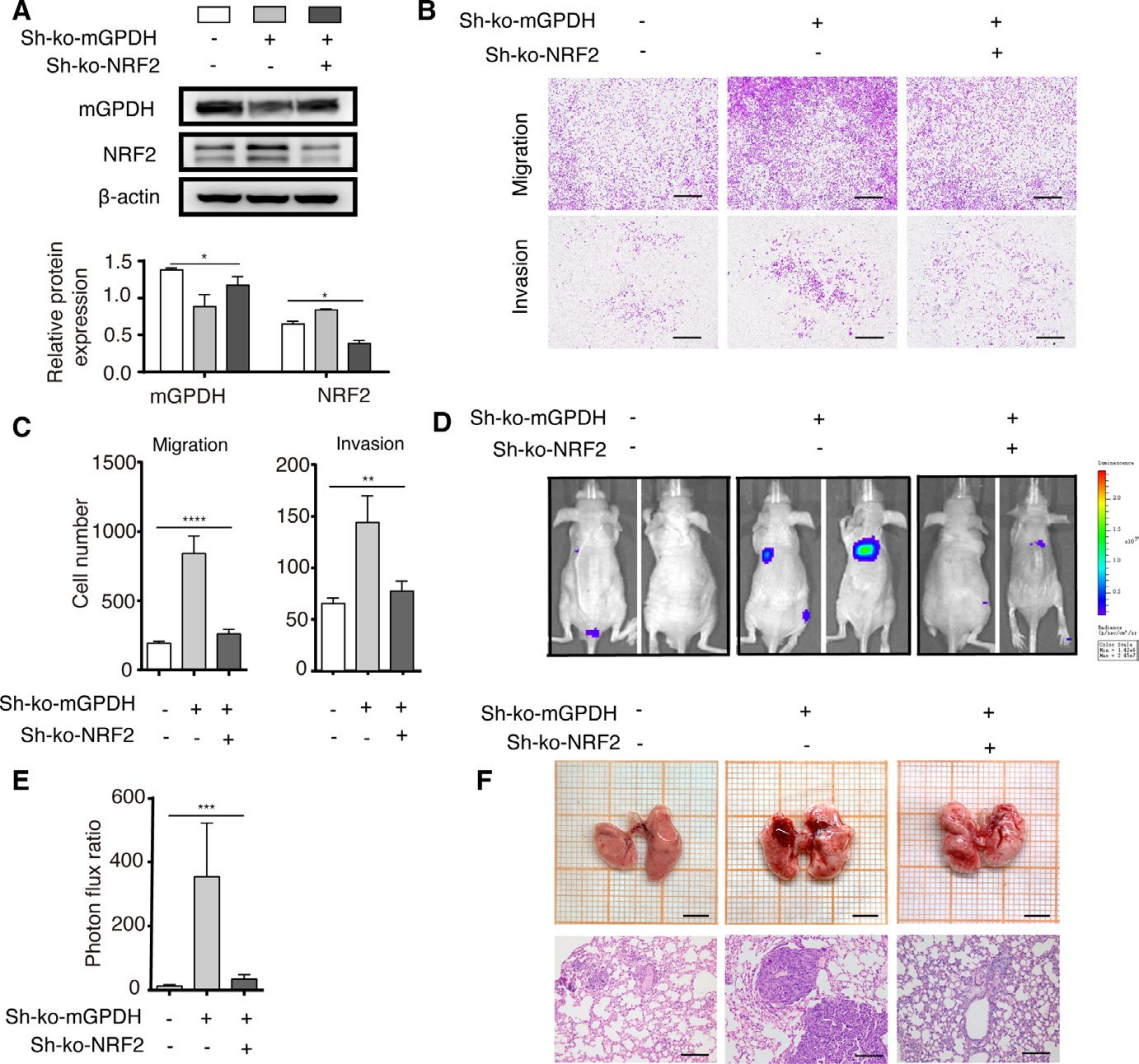
Down‐regulation of NRF2 rescued mGPDH loss‐induced melanoma metastasis. Control shRNA, mGPDH‐ko shRNA (Sh‐ko‐mGPDH) and/or NRF2 ‐ko shRNA (Sh‐ko‐NRF2) were transfected into A375 cells to construct the following 3 stable cell lines: Sh‐control, Sh‐ko‐mGPDH and Sh‐ko‐mGPDH Sh‐ko‐NRF2 A375 cells. These cell lines were cultured for 8 wk. A, WB analysis was used to detect mGPDH and NRF2 expression, and the intensity was quantified in the three cell lines. B, C, Cell migration and invasion were evaluated and quantified by Transwell migration and Matrigel assays, respectively (n = 3). Scale bar, 200 μm. D, In in vivo metastasis assays, nude mice were injected with the three stable cell lines. D, E, After 8 wk, melanoma cell metastasis in the nude mice was assessed by bioluminescence imaging and quantified as the photon flux ratio (n = 5 mice per group). F. Lung images and H&E staining for the three groups. Scale bars, 500 μm and 200 μm. The *P*‐Values were derived from one‐way ANOVA with the Newman‐Keuls multiple comparisons test. *P < 0.05, **P < 0.01, ***P < 0.001, ****P < 0.0001

To confirm the role of NRF2 in melanoma metastasis induced by the loss of mGPDH, we constructed stable A375 cell lines with decreased levels of both mGPDH and NRF2 by transfecting cells with mGPDH‐ko shRNA and NRF2‐ko shRNA (Figure 4A). In vitro migration and invasion assays were used to detect the metastatic ability of these A375 cells, and the mGPDH‐ko shRNA‐transduced A375 cells showed greater metastatic ability than the control shRNA‐transduced cells, while the mGPDH‐NRF2‐double ko group displayed numbers of migrated cells similar to those of the control group (Figure 4B,C), indicating that the loss of mGPDH accompanied by increased NRF2 expression contributed to A375 cell migration and invasion, while a genetic reduction in NRF2 levels rescued the melanoma phenotype to a relative normal phenotype. To confirm the metastatic process in vivo, nude mice were intravenously injected with the following three types of A375 cells: control shRNA, mGPDH‐ko shRNA, and mGPDH‐NRF2‐double ko A375 cells. After 8 weeks, bioluminescence imaging showed more metastases (Figure 4D), a higher photon flux ratio (Figure 4E) and worse histology and morphology (Figure 4F) in the lungs of mice injected with mGPDH‐ko shRNA A375 cells. An additional reduction in NRF2 converted these highly invasive mGPDH‐ko cells into weakly metastatic entities since the lungs of mice with the double‐ko A375 cell xenografts showed fewer haemorrhage spots and less consolidation (Figure 4D‐F). Therefore, both the in vivo and in vitro experiments demonstrated that the loss of mGPDH and increased downstream NRF2 in melanoma cells led to metastasis. For metastatic melanoma with a low mGPDH level, reduced NRF2 expression might be a target strategy to prevent melanoma from worsening.

### 3.5 Elevated mGPDH expression alleviated melanoma metastasis

**FIGURE 5:**
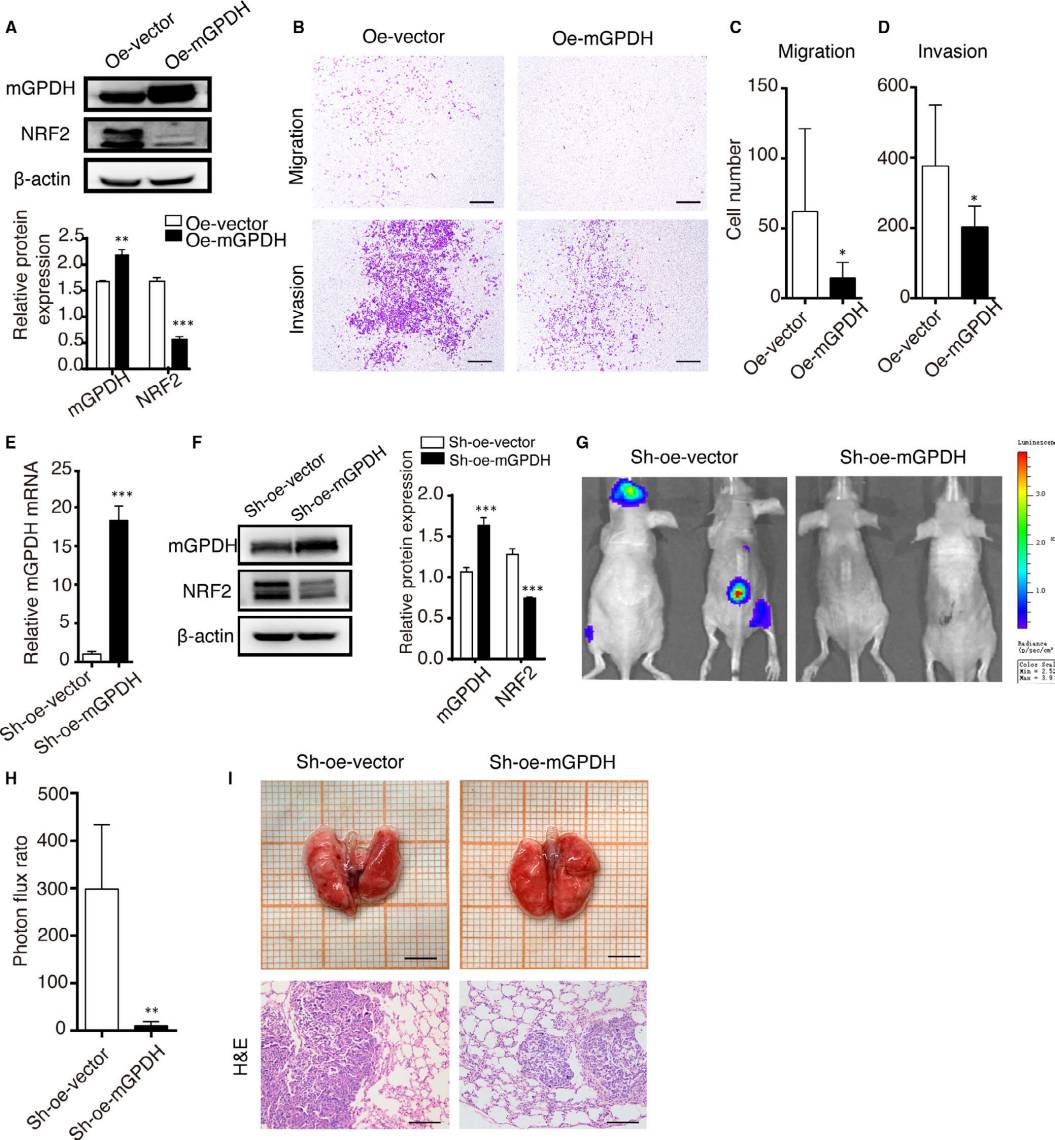
mGPDH‐overexpression alleviated melanoma metastasis. A‐D, A375 cells were transfected with a vector (Oe‐vector) or mGPDH‐overexpression plasmid (Oe‐mGPDH). A, Relative protein levels were detected by WB analysis, and the intensity was quantified. B, Transwell migration (upper) and Matrigel invasion assays (lower). Scale bar, 200 μm. C, D, Relative results of cell quantification by Transwell migration and Matrigel invasion assays (n = 3). E‐I, Luciferase‐control vector shRNA (Sh‐oe‐vector) or luciferase‐overexpression mGPDH shRNA (Sh‐oe‐mGPDH) was transfected into the A375 cell line to construct stable cell lines. These cells were intravenously injected into nude mice, and melanoma cell metastasis was observed for 8 wk in vivo. E, The relative mRNA expression of mGPDH was detected (n = 3). F, Transfected A375 cell lines were consistently cultured in vitro for 8 wk, and then mGPDH and NRF2 protein levels were detected. G‐I, In vivo metastasis assays. G, H, At 8 wk post‐injection of the stable cell lines, melanoma cell metastasis in the nude mice was assessed by bioluminescence imaging and quantified (n = 4 mice per group). I, Metastatic lung tissue images and H&E staining of the two groups. Scale bars, 500 μm and 200 μm. The *p‐*values were derived from Student's *t* tests. ***P < 0.001, **P < 0.01

Finally, the therapeutic effect of mGPDH overexpression on melanoma was evaluated. The mGPDH plasmid (Oe‐mGPDH) was transfected into A375 cells, which led to increased mGPDH protein levels in the A375 cells, accompanied by down‐regulated NRF2 (Figure 5A). As expected, an in vitro metastatic assay showed decreased migration and invasion abilities in the mGPDH plasmid‐transduced cells compared to the vector‐transduced control cells (Figure 5B‐D). Then, we constructed A375 cells stably expressing high levels of mGPDH by transducing the cells with mGPDH‐oe shRNA (Sh‐oe‐mGPDH). This stable cell line showed consistently higher mGPDH mRNA and protein levels, even after culture for 8 weeks, consistent with the in vivo duration (Figure 5E,F). Consistently, further in vivo experiments confirmed that increased mGPDH levels alleviated distant metastasis (Figure 5G,H) and improved the pathological phenotype of the xenografts (Figure 5I). According to the above experiments, enhanced mGPDH expression alleviated melanoma metastasis and further progression.

## 4 DISCUSSION

Melanoma is the most dangerous type of skin cancer and exhibits higher morbidity and mortality than other skin cancers. In the presence of distant metastasis, this cancer is generally considered incurable.^23^, ^24^ Redox homeostasis is a vital mechanism underlying melanoma oncogenesis and progression.^5^ However, further development is required for an efficient biomarker to guide gene therapy. In this study, we found that mGPDH was decreased in melanoma tissue, especially metastatic melanoma, which predicted a poor clinical outcome, and that the loss of mGPDH promoted metastasis by up‐regulating the NRF2 signalling pathway. Genetic inhibition of NRF2 rescued mGPDH ablation‐induced melanoma increases in migration and invasion. Correspondingly, mGPDH overexpression had anti‐metastatic effects on the melanoma cells, indicating its therapeutic potential.

Previous studies have demonstrated that mGPDH acts as a mitochondrial oxidative modulator and is usually expressed in low amounts in several cancers^25^, ^26^; however, the effect of decreased mGPDH expression in tumour progression has remained largely unknown. In this study, we report several findings regarding the role of mGPDH in melanoma. Specifically, mGPDH protein expression was decreased in primary melanoma tissue from patients, especially those with melanoma at a more severe stage or melanoma with lymph node metastasis, suggesting a negative correlation between mGPDH expression and melanoma progression. Moreover, in in vivo and in vitro xenograft experiments, the deletion of mGPDH by siRNA or shRNA aggravated melanoma cell migration and invasion, and the overexpression of mGPDH abrogated distant melanoma metastasis. These results confirm the effect of mGPDH in modulating melanoma metastasis. In addition, we did not find that mGPDH had obvious effects on melanoma cell proliferation, ATP production or apoptosis. However, Shilpa Thakur et al reported that mGPDH regulates thyroid cancer (follicular and papillary thyroid cancer) growth and metabolism.^8^ The unique and specific biological characteristics of different kinds of cancers might partially explain the differences in these results.^27^, ^28^

NRF2 senses oxidants and regulates antioxidant defence in melanoma metabolism,^16^, ^29^ which has been well proven to contribute to cancer progression.^4^ Our results confirm the vital role of NRF2 in the effect of decreased mGPDH on melanoma metastasis. First, not only is NRF2 a traditional tumour suppressor before tumorigenesis but also its hyperactivation has cancer‐promoting functions.^4^, ^29^ Numerous studies have revealed the mechanism underlying the role of excessive NRF2 activation in tumour metastasis.^30^ Comparably, our results demonstrate that NRF2 was over‐activated in skin tissues from patients with melanoma and that inhibiting NRF2 could rescue mGPDH loss‐induced melanoma distant metastasis. Second, mGPDH silencing in melanoma cells led to the up‐regulation of members of the NRF2 signalling pathway, including the transcript and protein levels of NRF2 and its downstream genes, but did not have a significant effect on NRF2 protein degradation. Consistently, recent studies have reported that NRF2 can be modulated at the protein and transcriptional levels.^21^, ^22^ Third, as in previous studies,^29^, ^31^ we also found that the inhibition of NRF2 improved the prognosis of melanoma. These results provide evidence supporting the pharmacological application of NRF2 inhibitors, such as ML385 and brusatol, for the prevention of distant melanoma metastasis and treatment of metastatic melanoma, although some deficiencies in these compounds remain.^32^

Regretfully, although the regulatory effect of NRF2 on cancer progression has been well‐established, few studies have comprehensively evaluated the effect of mGPDH on cancer. We focussed on melanoma metastasis through only a rough, preliminary tissue array screening. More studies are needed to explore the functions of mGPDH and the underlying molecular mechanism of NRF2 activation induced by decreased mGPDH expression. Regarding the clinical value and translational medicinal application of mGPDH, mGPDH could be a reference marker helpful in evaluating the risk of distant metastasis in patients with melanoma. Specifically, a lower mGPDH level indicated a more invasive melanoma phenotype. Novel therapies targeting the balance in mGPDH levels could also be investigated before its clinical use, which requires further research and development.

In summary, these findings confirm that mGPDH negatively regulates melanoma metastasis by modulating the NRF2 signalling pathway and provide several insights into mGPDH‐based targeted therapy to inhibit NRF2, which might be an attractive anti‐melanoma treatment approach in the future.

## CONFLICTS OF INTEREST

The authors have no potential conflicts of interest to declare.

## AUTHOR CONTRIBUTIONS

**Xing Li:** Formal analysis (equal); Investigation (lead); Methodology (lead); Software (equal); Writing‐original draft (lead); Writing‐review & editing (equal). **Ling Zhou:** Investigation (equal). **Yiming Zhang:** Investigation (equal). **Xuan He:** Investigation (equal). **Hao Lu:** Investigation (equal). **Linlin Zhang:** Methodology (equal); Validation (equal). **Yongfeng Tian:** Investigation (equal); Methodology (equal). **Xiufei Liu:** Investigation (equal); Methodology (equal). **Hongting Zheng:** Conceptualization (equal); Funding acquisition (equal); Supervision (equal). **Jiaqing Shao:** Supervision (equal); Writing‐review & editing (equal). **Min Long:** Funding acquisition (lead); Investigation (equal); Project administration (equal); Supervision (equal); Writing‐review & editing (equal).

## ETHICAL APPROVAL

This study was approved by the Medical Ethics Committee of the Second Affiliated Hospital of Army Medical University (No. 2018‐096‐01). All animal protocols were approved by the Army Medical University Institutional Animal Care and Use Committee. Our study was performed in accordance with the Declaration of Helsinki.

## Supporting information

Fig S1

Fig S2

Fig S3

Table S1‐3

Supplementary Material

## Data Availability

Data and material will be available from the corresponding authors.
