# Supplementary figures and images for: mGPDH Deficiency leads to melanoma metastasis via induced NRF2
Source: J Cell Mol Med. 2021 May 3;25(11):5305–15. doi: 10.1111/jcmm.16542 (PMC8178277; doi:10.1111/jcmm.16542)

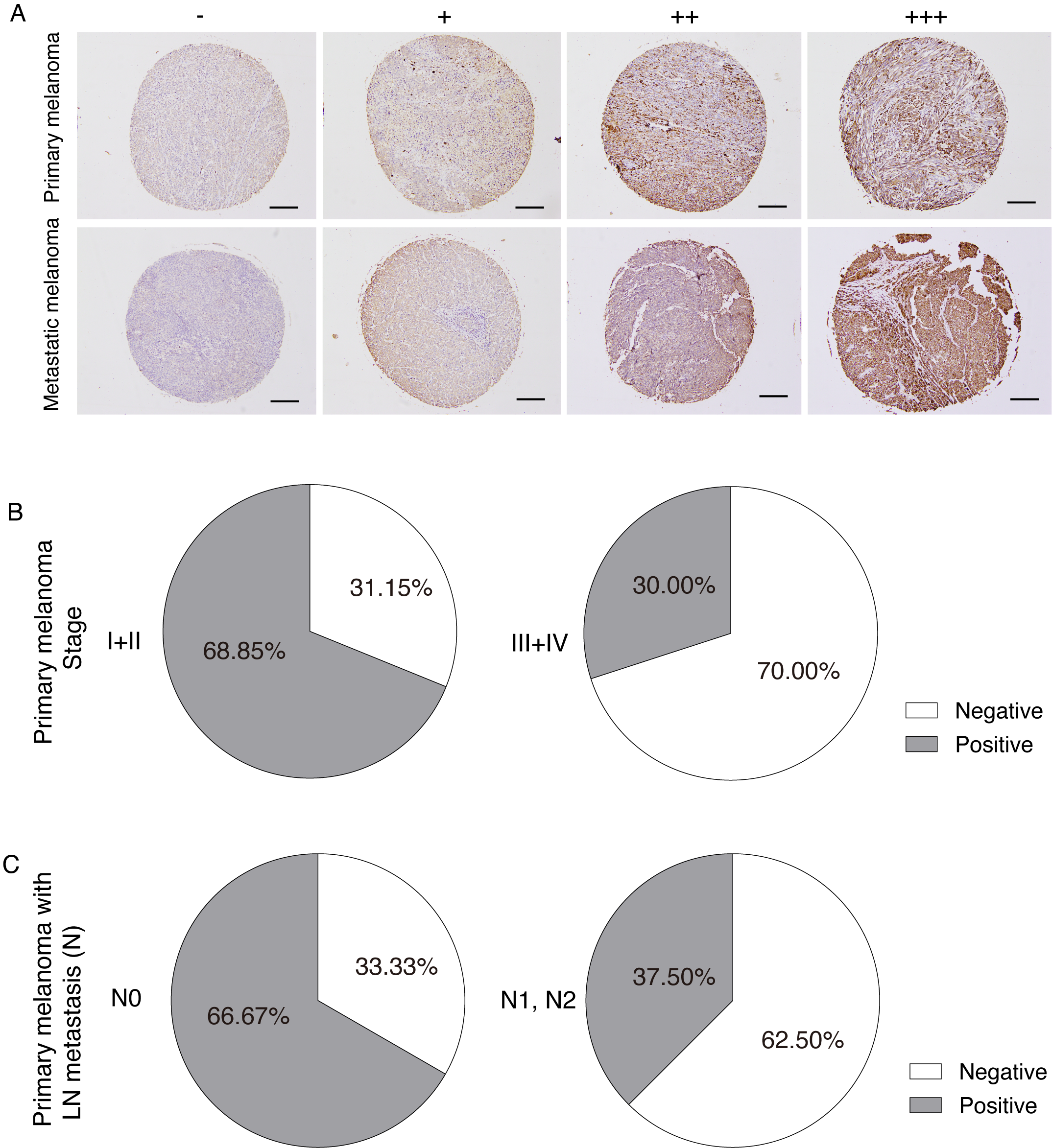

Supplement: Supplementary file 1 — Fig S1 [file JCMM-25-5305-s004.tif]

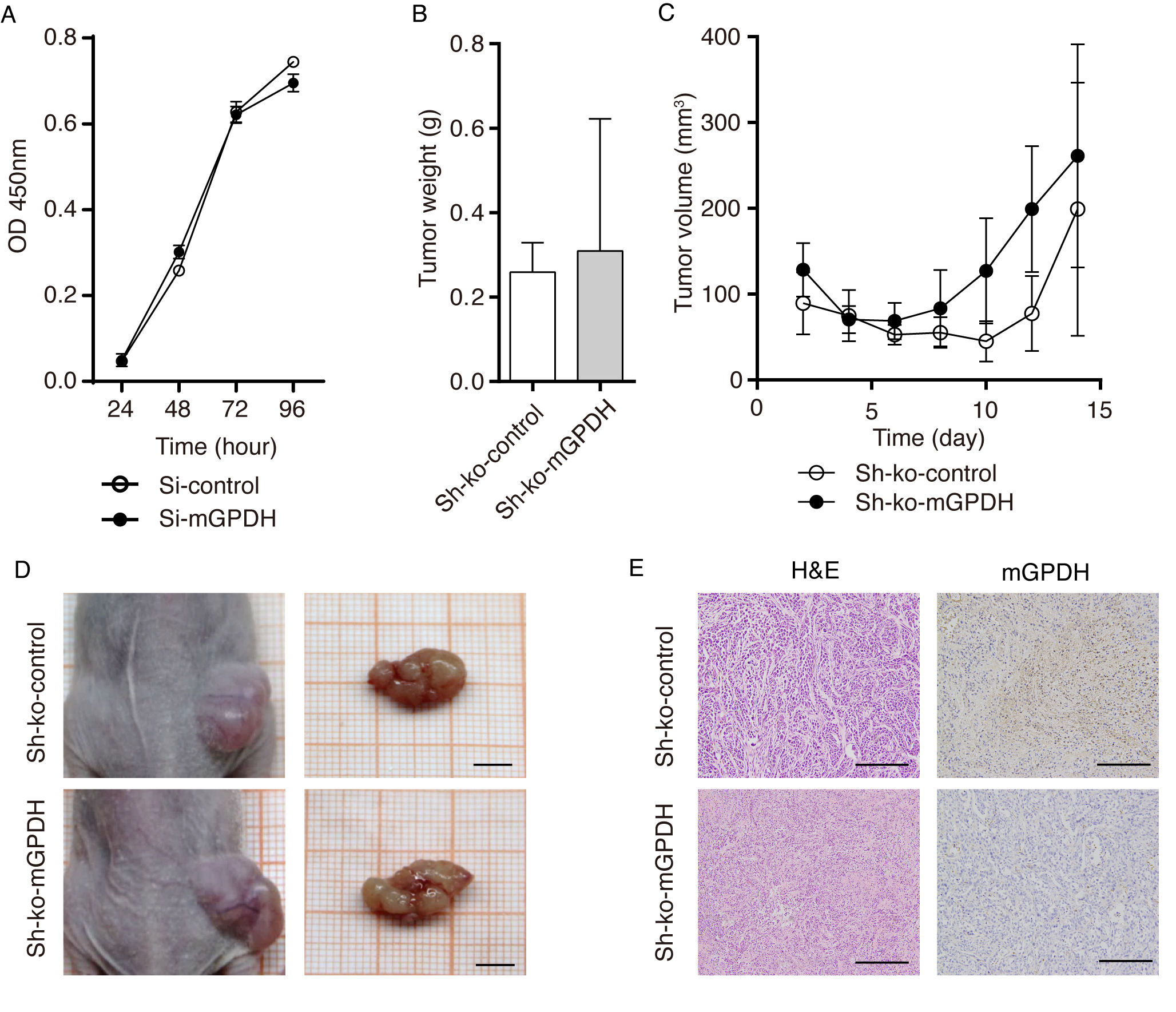

Supplement: Supplementary file 2 — Fig S2 [file JCMM-25-5305-s005.tif]

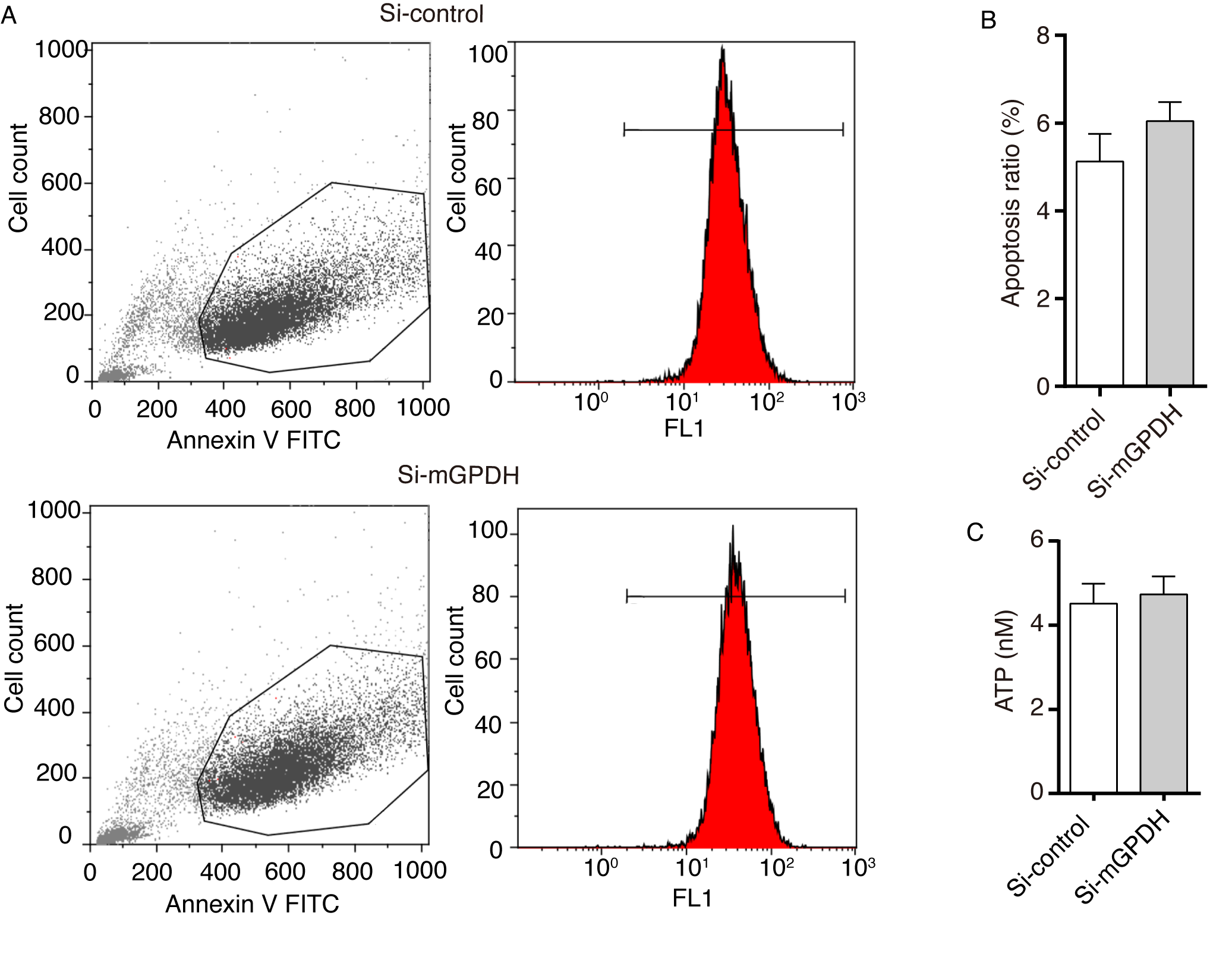

Supplement: Supplementary file 3 — Fig S3 [file JCMM-25-5305-s003.tif]
