# Supplementary material for: mGPDH Deficiency leads to melanoma metastasis via induced NRF2
Source: J Cell Mol Med. 2021 May 3;25(11):5305–15. doi: 10.1111/jcmm.16542 (PMC8178277; doi:10.1111/jcmm.16542)
Supplement: Supplementary file 4 — Table S1‐3 [file JCMM-25-5305-s001.docx]

Supplementary Table 1. Patients’ information of the tissue array and IHC scores of mGPDH.

| Age | Sex | Organ/Anatomic Site | Pathology diagnosis | TNM | Stage | Type | IHC score |
| --- | --- | --- | --- | --- | --- | --- | --- |
| 40 | M | Pleura | Malignant melanoma of right chest wall | T4N0M0 | IIB | Malignant | 3.4 |
| 70 | F | Oral cavity | Malignant melanoma of left parotid gland | - | - | Malignant | 6.4 |
| 64 | M | Esophagus | Malignant melanoma of esophagus | - | - | Malignant | 2 |
| 73 | M | Small intestine | Malignant melanoma of small intestine | - | - | Malignant | 4 |
| 71 | M | Small intestine | Malignant melanoma of small intestine | - | - | Malignant | 5.6 |
| 38 | F | Rectum | Malignant melanoma of rectum | - | - | Malignant | 0.8 |
| 42 | F | Rectum | Malignant melanoma of rectum | - | - | Malignant | 0.4 |
| 67 | M | Rectum | Malignant melanoma of rectum | - | - | Malignant | 0.6 |
| 67 | F | Rectum | Malignant melanoma of rectum | - | - | Malignant | 0.6 |
| 66 | F | Rectum | Malignant melanoma of rectum | - | - | Malignant | 1.6 |
| 44 | F | Rectum | Malignant melanoma of rectum | - | - | Malignant | 0.2 |
| 64 | F | Rectum | Malignant melanoma of rectum | - | - | Malignant | 0.2 |
| 75 | M | Rectum | Malignant melanoma of rectum | - | - | Malignant | 0.2 |
| 54 | F | Rectum | Malignant melanoma of rectum | - | - | Malignant | 1.2 |
| 52 | F | Rectum | Malignant melanoma of rectum | - | - | Malignant | 0 |
| 82 | F | Rectum | Malignant melanoma of anal canal | T4N0M0 | IIB | Malignant | 1.4 |
| 57 | M | Rectum | Malignant melanoma of rectum | - | - | Malignant | 0 |
| 84 | F | Rectum | Malignant melanoma of rectum | - | - | Malignant | 0 |
| 69 | F | Rectum | Malignant melanoma of rectum | - | - | Malignant | 0.2 |
| 64 | M | Rectum | Malignant melanoma of rectum | - | - | Malignant | 2.8 |
| 52 | F | Rectum | Malignant melanoma of anal canal | T4N0M0 | IIB | Malignant | 2 |
| 55 | M | Stomach | Malignant melanoma of stomach | - | - | Malignant | 1.6 |
| 55 | M | Stomach | Malignant melanoma of stomach | - | - | Malignant | 2.4 |
| 41 | F | Vulva | Malignant melanoma of cunnus | T4N0M0 | IIB | Malignant | 0 |
| 34 | F | Vulva | Malignant melanoma of cunnus | T4N0M0 | IIB | Malignant | 1.4 |
| 38 | F | Vulva | Malignant melanoma of cunnus | T4N0M0 | IIB | Malignant | 2 |
| 62 | F | Vulva | Malignant melanoma of vagina (sparse) | T4N0M0 | IIB | Malignant | 1.2 |
| 44 | F | Vulva | Malignant melanoma of cunnus | T4N0M0 | IIB | Malignant | 0.2 |
| 42 | F | Vulva | Malignant melanoma of cunnus (sparse) | - | - | Malignant | 0.8 |
| 57 | F | Vulva | Malignant melanoma of cunnus | T4N0M0 | IIB | Malignant | 5.2 |
| 79 | F | Vulva | Malignant melanoma of cunnus | T4N1M0 | III | Malignant | 1 |
| 44 | F | Cervix | Malignant melanoma of cervix | - | - | Malignant | 0.2 |
| 50 | F | Central nerve | Malignant melanoma of left brain | - | - | Malignant | 2.6 |
| 62 | F | Urethra | Malignant melanoma of urethra | T4N0M0 | IV | Malignant | 0.4 |
| 53 | F | Bladder | Malignant melanoma of bladder (sparse) | - | - | Malignant | 4.8 |
| 62 | M | Scrotum | Malignant melanoma of scrotum | T2BN0M0 | IIA | Malignant | 0.4 |
| 46 | M | Scrotum | Malignant melanoma of scrotum | - | - | Malignant | 1.2 |
| 38 | F | Nose | Malignant melanoma of left nasal cavity | - | - | Malignant | 0.4 |
| 56 | F | Nose | Malignant melanoma of right maxillary sinus | - | - | Malignant | 2.8 |
| 50 | M | Nose | Malignant melanoma of nose | - | - | Malignant | 0.2 |
| 63 | M | Nose | Malignant melanoma of nose | - | - | Malignant | 0 |
| 31 | M | Neck | Malignant melanoma of left neck | T4N1M0 | III | Malignant | 1.2 |
| 45 | F | Soft tissue | Malignant melanoma of left thigh | T2N0M0 | IB | Malignant | 2.2 |
| 88 | F | Skin | Malignant melanoma of left sole | T4N0M0 | II | Malignant | 2 |
| 49 | M | Soft tissue | Malignant melanoma of left thigh | T4N0M0 | IIB | Malignant | 3.2 |
| 65 | M | Skin | Malignant melanoma of right sole | T2BN0M0 | IIA | Malignant | 2.8 |
| 42 | F | Soft tissue | Malignant melanoma of right thigh | T4N0M0 | IIB | Malignant | 4 |
| 71 | M | Soft tissue | Malignant melanoma of right buttock | T4N0M0 | IIB | Malignant | 3.2 |
| 54 | M | Soft tissue | Malignant melanoma of left index finger | T4N0M0 | IIB | Malignant | 0.2 |
| 49 | F | Soft tissue | Malignant melanoma of back | T1N0M0 | I | Malignant | 0.2 |
| 42 | F | Skin | Malignant melanoma of left thumb | T4N0M0 | IIB | Malignant | 0.4 |
| 58 | F | Skin | Malignant melanoma of left buttock | T4N0M0 | IIB | Malignant | 4 |
| 50 | M | Skeletal muscle | Malignant melanoma of left shoulder | T4N0M0 | IIB | Malignant | 1.8 |
| 7 | M | Skeletal muscle | Malignant melanoma of sacrococcygeal region | T4N0M0 | IIB | Malignant | 5 |
| 66 | M | Skin | Malignant melanoma of sole | T2BN0M0 | IIA | Malignant | 2.2 |
| 57 | M | Skin | Malignant melanoma of left shoulder | T4N0M0 | IIB | Malignant | 7.2 |
| 52 | M | Skin | Malignant melanoma of abdominal part | T4N0M0 | IIB | Malignant | 8 |
| 56 | M | Skin | Malignant melanoma of abdominal wall | T4N0M0 | IIB | Malignant | 7.6 |
| 54 | F | Skin | Malignant melanoma of left heel | T4N0M0 | II | Malignant | 4 |
| 83 | F | Skin | Malignant melanoma of right little finger | T2N0M0 | IIA | Malignant | 4 |
| 36 | M | Skin | Malignant melanoma of back | T3N0M0 | II | Malignant | 6 |
| 38 | M | Skin | Malignant melanoma of left sole | T3N1M0 | III | Malignant | 7.6 |
| 49 | M | Skin | Malignant melanoma of left arm | T2BN0M0 | IIA | Malignant | 6.6 |
| 61 | F | Skin | Malignant melanoma of left foot | T4N0M0 | IIB | Malignant | 4.4 |
| 32 | F | Skin | Malignant melanoma of right lumbar part | T4N0M0 | IIB | Malignant | 3.2 |
| 41 | M | Skin | Malignant melanoma of left leg | T3N0M0 | IIA | Malignant | 6 |
| 51 | M | Skin | Malignant melanoma of back | T4N0M0 | IIB | Malignant | 3.2 |
| 72 | M | Skin | Malignant melanoma of left sole | T2N0M0 | I | Malignant | 7.6 |
| 63 | F | Skin | Malignant melanoma of right heel | T4N0M0 | II | Malignant | 1.2 |
| 62 | F | Skin | Malignant melanoma of right thumb | T4N0M0 | IIB | Malignant | 11.4 |
| 38 | F | Skin | Malignant melanoma of left upper arm | T1N0M0 | I | Malignant | 4.8 |
| 55 | M | Skin | Malignant melanoma of sole | T4N0M0 | II | Malignant | 1 |
| 51 | M | Skin | Malignant melanoma of chest wall | T4N0M0 | IIB | Malignant | 0.4 |
| 41 | F | Skin | Malignant melanoma of scalp | T4N0M0 | IIB | Malignant | 3.2 |
| 45 | M | Skin | Malignant melanoma of crissum | T4N0M0 | II | Malignant | 3.4 |
| 60 | M | Skin | Malignant melanoma of left sole | T4BN0M0 | IIC | Malignant | 5.4 |
| 71 | M | Skin | Malignant melanoma of right groin | T4N0M0 | IIB | Malignant | 1 |
| 66 | F | Skin | Malignant melanoma of chest wall | T4N0M0 | IIB | Malignant | 0 |
| 43 | F | Skin | Malignant melanoma of left leg | T2N0M0 | IIA | Malignant | 0 |
| 51 | M | Skin | Malignant melanoma of left armpit | T4N0M0 | IIB | Malignant | 6.6 |
| 36 | M | Skin | Malignant melanoma of right chest wall | T4AN0M0 | IIB | Malignant | 8.4 |
| 40 | M | Skin | Malignant melanoma of right chest wall | T4N0M0 | IIB | Malignant | 7.2 |
| 46 | F | Skin | Malignant melanoma of right chest wall | T4N0M0 | IIB | Malignant | 7.2 |
| 47 | F | Skin | Malignant melanoma of right upper arm | T4N0M0 | IIB | Malignant | 0.4 |
| 43 | F | Skin | Malignant melanoma of right abdominal wall | T4N0M0 | IIB | Malignant | 3.6 |
| 60 | M | Skin | Malignant melanoma of right buttock | T4N1M0 | III | Malignant | 0.8 |
| 69 | M | Skin | Malignant melanoma of sole | T2N0M0 | IIA | Malignant | 3.4 |
| 31 | M | Skin | Malignant melanoma of scalp | T4N0M0 | IIB | Malignant | 6 |
| 80 | F | Skin | Malignant melanoma of neck | T4N1M0 | III | Malignant | 6.4 |
| 51 | M | Skin | Malignant melanoma of left upper arm | T4N1M0 | III | Malignant | 7.2 |
| 80 | M | Skin | Malignant melanoma of right sole | T4N0M0 | IIB | Malignant | 6.6 |
| 42 | M | Skin | Malignant melanoma of left heel | T3N2M1 | IV | Malignant | 0 |
| 46 | F | Skin | Malignant melanoma of thigh | T4N1M0 | III | Malignant | 0 |
| 37 | M | Skin | Malignant melanoma of right upper arm | T4N0M0 | IIB | Malignant | 7.2 |
| 59 | F | Skin | Malignant melanoma of anus | T4N0M0 | IIB | Malignant | 8.2 |
| 52 | M | Eye | Malignant melanoma of right eye (sparse) | T4N0M0 | III | Malignant | 0 |
| 41 | F | Eye | Uveal melanoma | T1N0M0 | I | Malignant | 6 |
| 49 | F | Eye | Malignant melanoma of right eyeball | T2N0M0 | II | Malignant | 1 |
| 35 | F | Eye | Uveal melanoma | - | - | Malignant | 6.6 |
| 32 | F | Eye | Uveal melanoma | T4N0M0 | II | Malignant | 2.2 |
| 52 | M | Eye | Malignant melanoma of left eye | T2N0M0 | I | Malignant | 0.8 |
| 62 | M | Lymph node | Metastatic malignant melanoma of left neck | - | - | Metastasis | 0 |
| 68 | M | Lymph node | Metastatic malignant melanoma of neck | - | - | Metastasis | 0.2 |
| 68 | M | Lymph node | Metastatic malignant melanoma of right armpit | - | - | Metastasis | 0.2 |
| 62 | M | Lymph node | Metastatic malignant melanoma of left groin | - | - | Metastasis | 0.2 |
| 80 | F | Lymph node | Metastatic malignant melanoma of right armpit | - | - | Metastasis | 0.2 |
| 46 | M | Lymph node | Metastatic malignant melanoma of right groin | - | - | Metastasis | 0.4 |
| 57 | F | Lymph node | Metastatic malignant melanoma of right groin | - | - | Metastasis | 0.4 |
| 49 | M | Lymph node | Metastatic malignant melanoma of right neck | - | - | Metastasis | 0.4 |
| 65 | M | Lymph node | Metastatic malignant melanoma of groin | - | - | Metastasis | 0.6 |
| 43 | F | Lymph node | Metastatic malignant melanoma of groin | - | - | Metastasis | 0.6 |
| 60 | F | Lymph node | Metastatic malignant melanoma of right groin | - | - | Metastasis | 0.6 |
| 58 | F | Lymph node | Metastatic malignant melanoma of left groin | - | - | Metastasis | 0.6 |
| 49 | M | Lymph node | Metastatic malignant melanoma of cavitas pelvis | - | - | Metastasis | 0.6 |
| 61 | F | Lymph node | Metastatic malignant melanoma of right groin | - | - | Metastasis | 0.8 |
| 50 | M | Lymph node | Metastatic malignant melanoma of right parotid gland | - | - | Metastasis | 0.8 |
| 56 | F | Lymph node | Metastatic malignant melanoma of right groin | - | - | Metastasis | 1 |
| 42 | F | Lymph node | Metastatic malignant melanoma of groin | - | - | Metastasis | 1.2 |
| 52 | M | Lymph node | Metastatic malignant melanoma of left groin | - | - | Metastasis | 1.2 |
| 41 | F | Lymph node | Metastatic malignant melanoma of right groin | - | - | Metastasis | 1.2 |
| 38 | M | Lymph node | Metastatic malignant melanoma of left groin | - | - | Metastasis | 1.4 |
| 38 | M | Lymph node | Metastatic malignant melanoma of left armpit | - | - | Metastasis | 1.6 |
| 38 | M | Lymph node | Metastatic malignant melanoma of armpit | - | - | Metastasis | 1.6 |
| 39 | M | Lymph node | Metastatic malignant melanoma of left femur inferior segment | - | - | Metastasis | 1.6 |
| 49 | M | Lymph node | Metastatic malignant melanoma of left armpit | - | - | Metastasis | 1.8 |
| 68 | F | Lymph node | Metastatic malignant melanoma of right groin | - | - | Metastasis | 1.8 |
| 62 | M | Lymph node | Metastatic malignant melanoma of groin | - | - | Metastasis | 1.8 |
| 31 | F | Lymph node | Metastatic malignant melanoma of neck | - | - | Metastasis | 2 |
| 53 | F | Lymph node | Metastatic malignant melanoma of right groin | - | - | Metastasis | 2 |
| 70 | M | Lymph node | Metastatic malignant melanoma of armpit | - | - | Metastasis | 2.2 |
| 68 | F | Lymph node | Metastatic malignant melanoma of left groin | - | - | Metastasis | 2.2 |
| 48 | M | Lymph node | Metastatic malignant melanoma of neck | - | - | Metastasis | 2.2 |
| 63 | F | Lymph node | Metastatic malignant melanoma of right groin | - | - | Metastasis | 2.4 |
| 71 | M | Lymph node | Metastatic malignant melanoma of right armpit | - | - | Metastasis | 2.4 |
| 49 | F | Lymph node | Metastatic malignant melanoma of neck | - | - | Metastasis | 2.6 |
| 62 | M | Lymph node | Metastatic malignant melanoma of groin | - | - | Metastasis | 2.6 |
| 41 | F | Lymph node | Metastatic malignant melanoma of groin | - | - | Metastasis | 2.8 |
| 29 | F | Lymph node | Metastatic malignant melanoma of neck | - | - | Metastasis | 3 |
| 55 | F | Lymph node | Metastatic malignant melanoma of right occipital lobe | - | - | Metastasis | 3 |
| 55 | M | Lymph node | Metastatic malignant melanoma of left groin | - | - | Metastasis | 3.2 |
| 61 | M | Lymph node | Metastatic malignant melanoma of right groin | - | - | Metastasis | 3.5 |
| 40 | F | Lymph node | Metastatic malignant melanoma of right groin | - | - | Metastasis | 3.6 |
| 61 | F | Lymph node | Metastatic malignant melanoma of right leg | - | - | Metastasis | 3.6 |
| 44 | M | Lymph node | Metastatic malignant melanoma of right armpit | - | - | Metastasis | 3.8 |
| 49 | M | Lymph node | Metastatic malignant melanoma of left groin | - | - | Metastasis | 4.4 |
| 60 | F | Lymph node | Metastatic malignant melanoma of neck | - | - | Metastasis | 4.8 |
| 56 | F | Lymph node | Metastatic malignant melanoma of groin | - | - | Metastasis | 5.6 |
| 45 | M | Lymph node | Metastatic malignant melanoma of right armpit | - | - | Metastasis | 5.6 |
| 41 | F | Lymph node | Metastatic malignant melanoma of groin | - | - | Metastasis | 5.6 |
| 45 | F | Lymph node | Metastatic malignant melanoma of left chest wall | - | - | Metastasis | 6.2 |
| 70 | M | Lymph node | Metastatic malignant melanoma of groin | - | - | Metastasis | 6.4 |
| 56 | M | Lymph node | Metastatic malignant melanoma of left preauricula | - | - | Metastasis | 6.4 |
| 65 | M | Lymph node | Metastatic malignant melanoma of right groin | - | - | Metastasis | 6.6 |
| 47 | F | Lymph node | Metastatic malignant melanoma of armpit | - | - | Metastasis | 8.4 |

Supplementary Table 2: siRNA and shRNA sequences.

| Target | Sequence |
| --- | --- |
| Si-mGPDH | TTCTCCGAACGTGTCACGTAA |
| Sh-ko-mGPDH | GATCCGTTCTCCGAACGTGTCACGTAATTCAAGAGATTACGTGACACGTTCGGAGAATTTTTTC |
| Sh-ko-NRF2 | CCGGCATTTCACTAAACACAACTCGAGTTGTGTTTA GTGAAATGCCGGTTTTTG |
| Oe-mGPDH plasmid | NCBI Reference Sequence: NM_000408.5 |
| Sh-oe-mGPDH | NCBI Reference Sequence: NM_000408.5 |

Supplementary Table 3: Primer sequences.

| Primer Target | 5’ | 3’ |
| --- | --- | --- |
| H-mGPDH | CGGACAACATAACGATGCAC | CTGTCTGGGGGTCTGTCTTC |
| H-NRF2 | CGACGTGTGGCGGCTGAG | GCTGCTGTGACGGCCAACC |
| H-HO1 | GAGGAGTTGCAGGAGCTGCT | GAGTGTAAGGACCCATCGGA |
| H-GAPDH | CTCCTCCTGTTCGACAGTCAGC | CCCAATACGACCAAATCCGTT |
