# Supplementary material for: mGPDH Deficiency leads to melanoma metastasis via induced NRF2
Source: J Cell Mol Med. 2021 May 3;25(11):5305–15. doi: 10.1111/jcmm.16542 (PMC8178277; doi:10.1111/jcmm.16542)
Supplement: Supplementary file 5 — Supplementary Material [file JCMM-25-5305-s002.docx]

Supplementary Figure 1. mGPDH expression in human melanoma tissue. A commercial melanoma tissue array (ME2082c, Biomax) was used, and the mGPDH expression levels were detected by IHC. A. Representative IHC images showing mGPDH expression in primary and metastatic melanoma tissue sections. Scale bar, 500 μm. B. The mGPDH IHC score distribution in primary melanoma with tumours at the following stages: stages I and II (the melanoma is confined to the skin), n=61; stages III and IV (the melanoma has spread), n=10. C. The distribution of the mGPDH IHC scores in primary melanoma tissues without or with lymph node metastasis: stage N0, n=63; stages N1 and N2, n=8. Negative: IHC score (-): 0-2. Positive: IHC score >2.

Supplementary Figure 2. mGPDH silencing did not influence melanoma proliferation *in vivo* or *in vitro*. Control shRNA (Sh-ko-control) and mGPDH-ko shRNA (Sh-ko-mGPDH) were transfected into A375 cells to construct stable melanoma cell lines. A. Proliferation assay with A375 cells transfected with control siRNA (Si-control) and mGPDH siRNA (Si-mGPDH) (n=3). B-E. These stable cell lines were subcutaneously injected into the backs of nude mice to construct an *in situ* melanoma model (n=6). Tumour growth was observed for 2 weeks. At the end of the experiment, the mice were sacrificed, and subcutaneous tumour tissues were harvested and analysed. B, C. Tumour weight and tumour volume in the nude mice. D. Representative tumour images. Scale bar, 500 μm. E. H&E and mGPDH IHC staining of the tumour tissues. Scale bar, 200 μm. The *p*-values were derived from Student’s *t* tests.

Supplementary Figure 3. mGPDH deficiency did not affect melanoma cell apoptosis or ATP production. A. Representative images showing the number of apoptotic cells determined by flow cytometry. B. Annexin V-FITC detection of the apoptosis ratio (%) in A375 cells transfected with control siRNA and mGPDH siRNA (n=3). C. ATP assay of A375 cells transfected with control siRNA and mGPDH siRNA (n=3). The *p*-values were derived from Student’s *t* tests.
